# Patents: unite to conquer

**DOI:** 10.1590/1516-3180.2013.1316770

**Published:** 2013-12-01

**Authors:** Alessandro Wasum Mariani, Paulo Manuel Pêgo-Fernandes

**Affiliations:** I MD. Thoracic Surgeon, Instituto do Coração (InCor), Hospital das Clínicas (HC), Faculdade de Medicina da Universidade de São Paulo (FMUSP), São Paulo, Brazil.; II MD, PhD. Associate Professor, Discipline of Thoracic Surgery, Instituto do Coração (InCor), Hospital das Clínicas (HC), Faculdade de Medicina da Universidade de São Paulo (FMUSP), São Paulo, Brazil.

The last 20 years have been important for Brazilian science. We have moved from a position close to obscurity to achieve significant status internationally. The most forceful proof of this is the great evolution of the number of published papers and citations achieved by Brazilian authors and institutions. The efforts made by government, universities, research centers and researchers have borne fruit measurable in terms of the number of published articles produced in Brazil. The latest information available demonstrates that in 2011, we reached a total number of 49,664 articles, which has made us leap up to 13th place in the ranking of countries with the highest numbers of published papers.^1^ On the other hand, it can be asked whether the impact of these published papers continues to be less than what would be expected. Nonetheless, one important point in this regard is the observation that several Brazilian universities are in a prominent position in the Webometrics "Ranking Web of Universities", particularly in relation to "impact".^2^

However, it is notable that another important facet of scientific activity has not kept up with this evolution: the production of patents. A study has demonstrated that the total number of patents applied for in Brazil between 2001 and 2010 increased by 64%, but the final number, which was 5,500 in 2010, is still small in the worldwide context.^3^ Another negative point is that, among the emerging economies of the world, Brazil still has a position of little significance, considering the number of patents developed here and how many of these end up generating a final marketable product. In 2011, just over 20,000 patents were registered in Brazil, which was a small number in comparison with China's production, which was 400,000 in the same year.^3^

According to the University of São Paulo's Innovation Agency, the notion of a patent is "...a temporary title of ownership over an invention or model of use that is granted by the State to the inventors or other physical individuals or legal entities that hold rights over the creation".^4^ The rationale and justification for the existence of patients lies in the fact that: "research and development for preparing new products generally requires large human and financial investments. Protecting these products through a patient signifies guarding against competitors and thus inhibiting unfair competition".^4^

Some important remarks can be made: 

Patents are justified by the products that they can generate. They are not titles, nor can they be regarded only as curricular items. 

The biggest potential beneficiary from patent production is Brazil as a country. After all, in the final analysis, patent production is a positive insertion into the Brazilian production matrix. 

In developed countries, a "virtuous circle" that maintains an active "system" of patent production can clearly be identified: companies inject money into research centers that develop projects, which are transformed into patents for the company, which then manufactures and markets the invention as a final product, thereby generating capital, which is often reinvested in the starting point. Government participation is not limited only to regulation, but intervention should also occur when this becomes necessary.

Coming back to Brazil, some positive initiatives in this regard can be identified. For example, the Research Support Foundation of the State of São Paulo (Fundação de Amparo à Pesquisa do Estado de São Paulo, Fapesp) has created new Centers for Research, Innovation and Dissemination (Centros de Pesquisa, Inovação e Difusão, Cepids). One of the characteristics of these centers is that they seek out and consolidate partnerships with production sectors with the final objective of promoting transformation to a culture of innovation.^5^

Another important initiative has come from the universities themselves, through creation of agencies with the common aim of providing support for innovation, either within the university or even outside of it, through partnerships with companies, researchers and research centers. Notably, the focus of these so-called "Innovation Agencies" is on developing patents. Several important universities have already taken this idea on board: University of São Paulo (Universidade de São Paulo, USP), State University of Campinas (Universidade Estadual de Campinas, Unicamp), São Paulo State University (Universidade Estadual Paulista, Unesp), Federal University of São Carlos (Universidade Federal de São Carlos, UFSCar), State University of Londrina (Universidade Estadual de Londrina, UEL) and Federal University of Rio de Janeiro (Universidade Federal do Rio de Janeiro, UFRJ), among others.

Government, universities and companies need to join forces and, especially, coordinate their efforts better, so that the activities of each segment complement each other. In this way, a production process that generates patents can be started, thus giving rise to leverage not only within science but also within the present production chain of the Brazilian economy.
